# Outcome of cataract surgery in children with presumed trematode-induced granulomatous anterior uveitis

**DOI:** 10.1186/s12886-023-03273-w

**Published:** 2024-01-15

**Authors:** Mona Abdallah, Ashraf K. Al-Hussaini, Wael Soliman, Mohamed G. A. Saleh

**Affiliations:** Department of Ophthalmology, Faculty of Medicine, Assiut University, Assiut, 71515 Egypt

**Keywords:** Cataract, Trematode, Uveitis

## Abstract

**Purpose:**

To examine the 6-month visual outcomes and complications following cataract surgery in patients with persumed trematode induced granulomatous anterior uveitis.

**Setting:**

Assiut university hospital, Assiut, Egypt.

**Design:**

This is a retrospective non comparative case series study**.**

**Methods:**

Patients presenting with significant cataract secondary to uveitis caused by trematode induced anterior chamber granuloma were included in this study.

Cases with active anterior uveitis, within the last 3 months preceding surgery, and those with a history of trauma, were excluded from this study.

Data collected included demographic characteristics, history of the condition including when uveitis started, treatment received and history of other health conditions that may be relevant to uveitis.Complete opthalmologic examination including assessment of best corrected visual acuity (BCVA) and OCT macula, if possible, were done. These was repeated 1 week, 1 month, 3 months and 6 months after surgery. Specular microscopy was performed preoperatively and 3 months after surgery. Patients underwent cataract surgery with posterior chamber intra ocular lens and statistical analysis was performed to compare preoperative and postoperative BCVA and corneal endothelial cell counts. Postoperative complications were recorded.

**Results:**

Five eyes of 5 patients were included in the study. All study eyes showed improvement in the post-operative visual acuity.

A statistically significant improvement was observed in VA in the sixth postoperative month compared to the baseline measurements (*p* = 0.004). No statistically significant difference was observed between the preoperative and postoperative endothelial cell counts (*p* = 0.696). Cystoid macular edema did not occur as a postoperative complication.

**Conclusion:**

Visual outcomes of cataract surgery in eyes with persumed trematode induced granulametous anterior uveitis are favorable. No sight threatening complication was observed in our series.

## Introduction

Parasitic infestation of ocular structures can cause significant intraocular inflammation with subsequent complications. Presumed trematode-induced uveitis is a distinct clinical entity, most frequently observed in children. Granulomatous inflammation that usually presents as one or more pearl-like nodules in the anterior chamber (AC) is the hallmark of this condition. Less frequently, subconjunctival lesions or corneal lesions can also occur [1–4]. In rare instances, intermediate uveitis with snow banking and posterior uveitis could be the presenting feature of this disease [5]. Children who swim in rivers harboring trematode-infected snails have a high risk of infestation by several waterborne trematodes [6]. Ocular inflammation may be caused by the tissue damage by the parasite and its toxic product or secondary to the reaction by the immune system of the host [7]. Procerovum varium, a trematode, was identified as the causative agent responsible for AC granuloma in children in South India [8, 9]. Schistosomiasis, also known as bilharziasis, is an endemic disease caused by trematodes of the Schistosoma genus in Egypt. relatively rare ocular involvement has been attributed to Bilharziasis, mainly in the form of granuloma reaction induced by the ova or adult worms in different ocular tissues. The first case of bilharzial conjunctival granuloma was reported in Egypt by Sobhy in 1928. A myriad of ocular lesions including keratitis, chronic conjunctivitis, chronic uveitis with complicated cataract, vitreous opacities, posterior uveitis in the form of chorioretinitis, subretinal granulomas, and pre-retinal hemorrhage was reported in the literature [10, 11]. Schistosoma mansoni was also observed and isolated from the AC angle [12]. There is a significant association between the the patients' residencies close to ponds and snail habitats and this condition of granulomatous reactions [13]. The cercariae (infective stage) of the trematode can reach maturity and lay ova directly in the veins of the richly vascularized conjunctiva, which subsequently leads to the development of sub-conjunctival granuloma. In some cases, cercariae can penetrate limbal structures and gain access to the anterior chamber leading to the development of anterior chamber granuloma with or without granulomatous uveitis. This has been considered the most accepted theory that explains how the ova of the trematode or adult worms reach the eye [10, 14, 15]. Many treatment modalities for the treatment of AC granulomas were reported, including topical corticosteroids which is considered a the standard line of treatment. Resistant cases may be treated with oral prednisone starting at a daily dose of 1 mg/kg which is gradually tapered over 3–6 weeks [16]. Therefore, many cases may develop steroid-related complications such as cataracts and glaucoma [17, 18]. In addition, persistent intra-ocular inflammation may contribute to development of cataract in those cases [18] This study aims at evaluation of the outcome of cataract surgery in childern with persumed trematode -induced granulomatous anterior uveitis.

## Patients and methods

All study procedures adhered to the tenets of the Declaration of Helsinki. Approval of our review was obtained from the IRB of the Faculty of Medicine, Assiut University (IRB No. 17200420). The parents of study subjects provided written informed consent before acquisition of the data of study subjects.

A retrospective chart review of the patients who presented to the uveitis service at outpatients clinic of the department of Ophthalmology, Assiut University hospital, Egypt between December 2020 and December 2021 with cataracts secondary by presumed trematode AC granuloma and underwent cataratct surgery was performed. The inclusion criterion was childern less than 15 years old with controlled uveitis for at least three months before cataract surgery.

Exclusion criteria were active anterior uveitis within the three months before enrollment in the study,and evidence of trauma. Also, patients with other ocular pathology that could influence the final visual acuity (VA), such as macular scars, glaucomatous optic nerve changes, and central corneal opacity, were excluded.

**Fig. 1:**
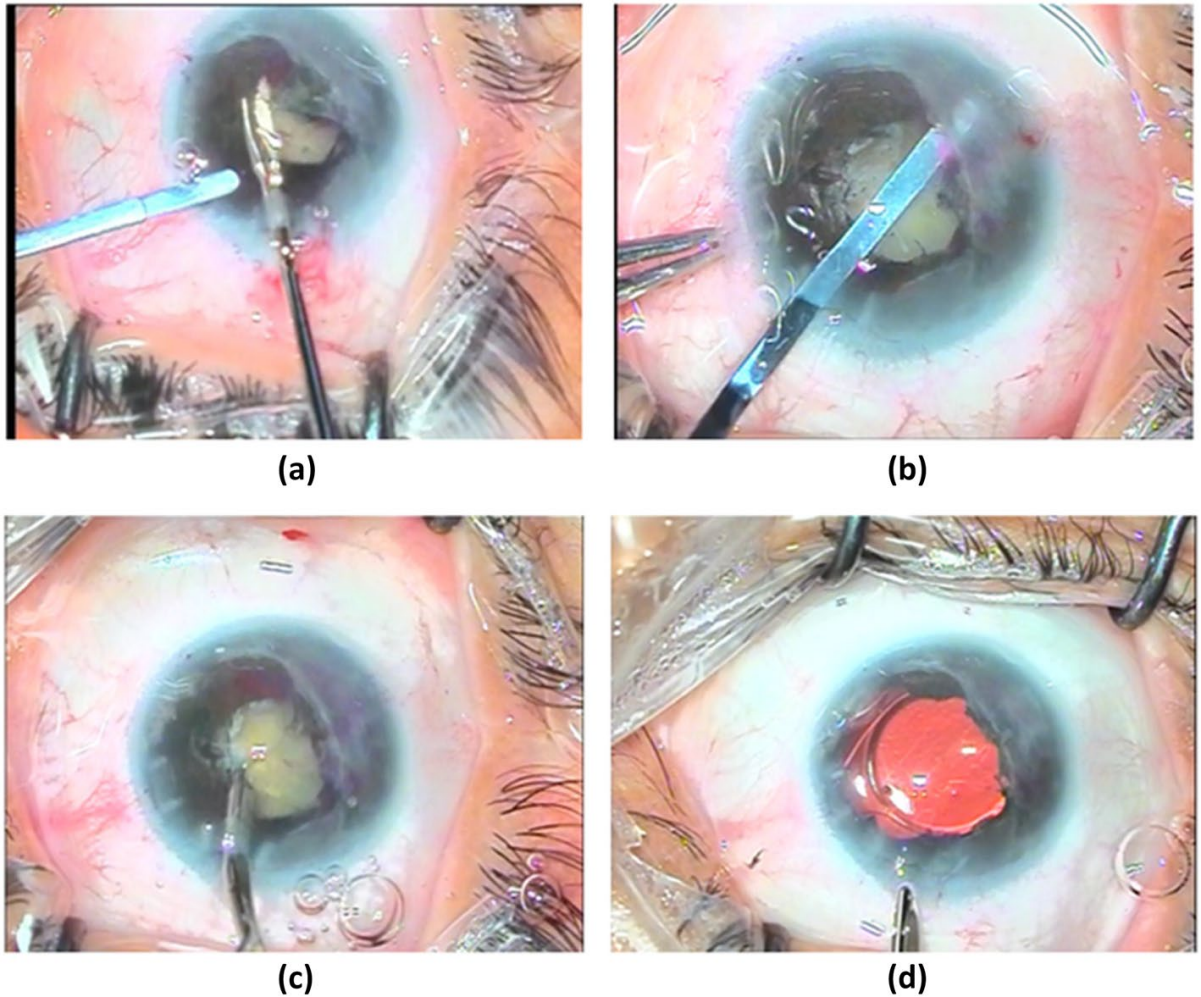
Steps of cataract surgery in a patient with trematode uveitis (**a**, **b**) synecholysis, (**c**) irrigation aspiration, and (**d**) implantation of the IOL

**Fig. 2:**
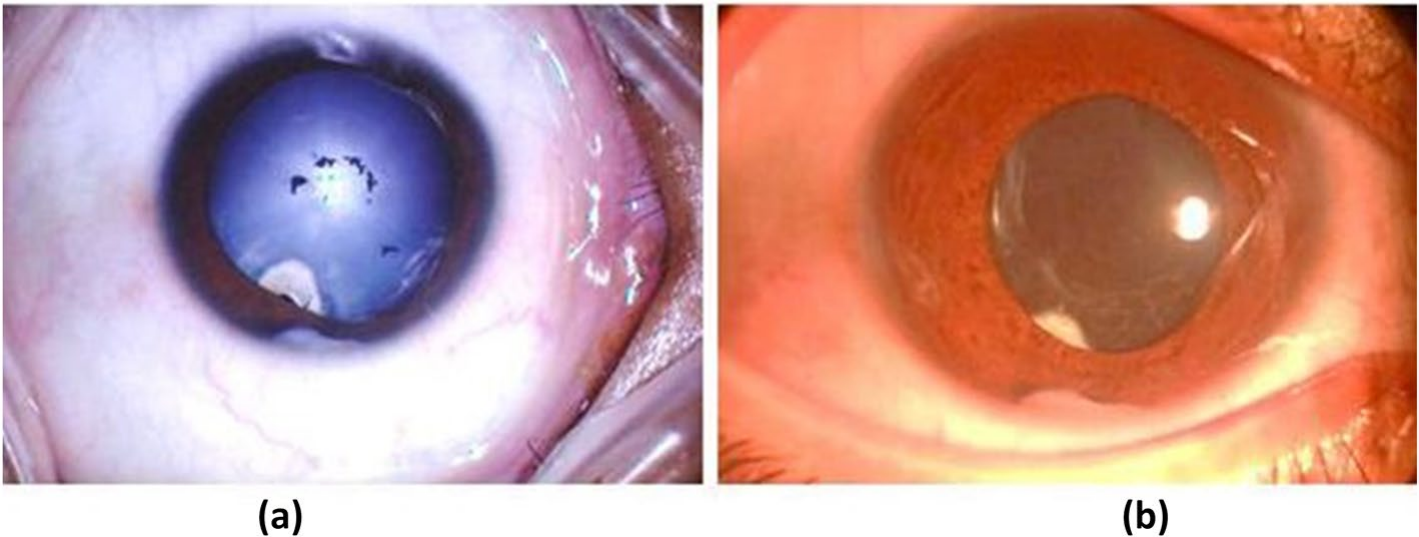
(**a**) preoperative cataract in a patient with trematode uveitis (**b**) after I/A of cataract, and implantation of the IOL

Data collected from the patients included the demographic characteristics, medical and ocular history, and the results of complete baseline ocular findings, including measurment of best corrected visual acuity (BCVA) using Snellen's chart, which was converted to logarith of minimum angle of resolution (logMAR) for statistical analysis, slit lamp anterior segment examination (including cornea, conjunctiva, eyelids and AC), intraocular pressure measurement, fundus examination, and optical coherence tomography (OCT) of the macular area if it was performed before surgery if the ocular media were sufficiently clear to obtain an adequate OCT signal. Pre-operative control of uveitis for at least three months was achieved using topical, peri-ocular, systemic steroids, or a combination of these. Intraocular lens (IOL) calculation was performed using IOL master by utilizing multiple IOL calculation formulas. Specular microscopy was performed to evaluate the corneal endothelial status. Surgical technique employed started by creating a 2.4-mm clear corneal incision using a microkeratome and two side port incisions using an MVR blade. Subsequently anterior chamber (AC) was filled with an ophthalmic viscoelastic device (OVD). This was followed by manual stretching and dilatation of the pupil using two push–pull instruments and peeling of the iris and pupillary membranes by capsulorhexis forceps. Trypan blue dye was injected and the excess dye was washed off in cases with dense white cataracts to allow visualization of the anterior lens capsule during the creation of the continuous curvilinear capsulorhexis (CCC), which was started and completed using the capsulorhexis forceps. Irrigation aspiration of soft lens and implantation of hydrophobic or hydrophilic IOL into the capsular bag was performed (Fig. 1). Subconjunctival injection of dexamethasone was done at the conclusion of surgery. Postoperative medications included topical moxifloxacin five times a day, topical cycloplegic mydriatic thrice daily, and difluprednate five times a day for one week with gradual tapering postoperatively. Topical fluromethanole was administered twice daily for one month, and systemic steroid 1 mg/kg was administered for one week followed by gradual tapering. Postoperative follow-up of those patients included measurement of BCVA, slit lamp examination incuding fundus examination, and intraocular pressure measurement using a handheld rebound tonometer. Assessment was repeated at one week, one month, three months, and six months postoperatively (Fig. 2).

### Statistical analysis

Data were collected in an Excel spreadsheet and analyzed using SPSS statistical software version 22 (IBM, Chicago, IL). The changes in the variables were compared with that at baseline using paired T-test.

## Results

### Demographic and baseline characteristics

Study subjects had an average age of 12 ± 1.58 years (range, 10–14 years), all patients were boys with unilateral involvement, and all resided in rural villages around Assiut city. All participants had a positive history of swimming in local ponds and canals and developed uveitis after exposure. The duration of symptoms was 22 ± 13 days, and the duration between the control of uveitis and cataract surgery was 5 ± 4.3 months.The demographic and baseline characteristics are summarized in Table 1.

**Table 1:** Demographic data and baseline charactaristics

Mean age	12 ± 1.58
Mean duration of symptoms	22 ± 13 days
Mean time between the control of uveitis and surgery	5 ± 4.3 months
Mean preoperative V/A	2.4 ± 0.894
Mean preoperative IOP	18.4 ± 2.7
Mean preoperative endothelial cell count	3003 ± 154.6

### Visual acuity

The mean preoperative BCVA represented as log MAR was 2.4 ± 0.894 log MAR, and the mean value of the 6-month postoperative BCVA was 0.22 ± 0.192 log MAR. A statistically significant improvement was observed in VA in the sexth postoperative month compared with the baseline measurements (*p* = 0.004) as mention in Tables 2 and 3. The mean value of the postoperative spherical equivalent was 0.2 ± 0.64.

**Table 2:** Comparison of the preoperative and postoperative visual acuity

Case Number	Preoperative BCVA logMAR	Postoperative BCVA logMAR at 6 months
1	** + 3.0**	**0.5**
2	** + 3.0**	**0.3**
3	** + 2.0**	**0.1**
4	** + 1.0**	**0.00**
5	** + 3.0**	**0.2**

**Table 3:** Statistical analysis of the preoperative and postoperative visual acuity: Paired samples statistics

		Mean	N	Std. Deviation	Std. Error Mean
Pair 1	Preoperative VA	2.4000	5	.89443	.40000
	Postoperative VA	.2200	5	.19235	.08602

### Endothelial cell count

The mean value of the endothelial cell count at baseline was 2928 ± 214, and the mean value of the 3-month postoperative endothelial cell count was recorded in 4 patients and was 2817 ± 458. No statistically significant difference was observed between the preoperative and postoperative endothelial cell counts (*p* = 0.696).

Changes in endothelial cell count are summarized in Table 4.

**Table 4:** Statistical analysis of the preoperative and postoperative endothelial cell count

Paired samples statistics									
		**Mean**		**N**	**Std. Deviation**	**Std. Error Mean**			
**Pair 1**	**Preoperative ECC**	**2928.2000**		**4**	**214.66299**	**96.00021**			
	**Postoperative ECC**	**2817.2000**		**4**	**458.34779**	**204.97936**			
**Paired samples test**									
		**Paired Differences**					**t**	**d f**	**Sig. (2-tailed)**
		**Mean**	**Std. Deviation**	**Std. Error Mean**	**95% Confidence Interval of the Difference**				
					**Lower**	**Upper**			
**Pair1**	**Preoperative ECC** **Postoperative ECC**	**111.00000**	**590.56541**	**264.10888**	**-622.28380-**	**844.28380**	**.420**	**4**	**.696**

### Central macular thickness (CMT)

The mean value of the postoperative CMT was 259.75 ± 32.26, indicating cystoid macular edema did not occur as a postoperative complication.

### Posterior capsular opacification (PCO)

PCO was observed in 2 patients. Reactivation of uveitis was reported in 2 patients one month postoperatively as a trace of cell and a mild flare and cell + 1.0 were observed in one patient each.

We summarized details of all five patients in the study as mentioned in Table 5.

**Table 5:** Summary of data of individual study subjects

Subject No	Age (years)	Duration of uveitis control before cataract surgery	Pre op IOP (mmHg)	Pre op BCVA logMAR	Post op BCVA logMAR	Pre op ECC	Post op ECC	Post op CMT (µm)
1	**13**	1 year	18	3.0	0.1	2861	2923	293
2	**12**	6 months	16	3.0	0	3082	3234	273
3	**14**	3 months	17	1.0	0.3	2888	2870	217
4	**11**	3 months	20	2.0	0.2	3182	3023	256
5	**10**	3 months	21	3.0	0.5	2628	2036	257

## Discussion

Granulomatous anterior uveitis in children is presumed to be caused by a trematode infections, especially in rural areas. There is a significant risk associated with swimming in unsanitary local ponds or rivers, as reported by several studies [1, 3, 5, 7, 19]. Cataract is the main complication in patients with uveitis and the leading cause of vision loss. Intraocular inflammation was considered a significant risk factor for cataract surgery [20].

All patients were males in our analysis due to cultural reasons, as it is rare to allow girls swimming in rivers or in public. All patients were below 15 years of age. All cases showed unilateral involvement with only a single nodule at the lower portion of AC (at the 6 o'clock position). This condition is reported to have a high incidence in Egypt, accounting for 22.2% of anterior uveitis cases in a study from Egypt.^4^ All cases in this study showed significant diminution of vision secondary to the presence of complicated cataract (the mean preoperative VA was 2.4 ± 0.894 Log MAR); however, in other studies, diminution of vision secondary to increased corneal thickness, flare, and cells ( VA 0.3 Log MAR) [21] This could be attributed to different inclusion criteria as we included patients after control of active uveitis. Objectively, all patients showed significant improvement in the postoperative VA (0.22 ± 0.647LogMAR) (*P* < 0.001).

Other studies have shown results similar to the present study. There is a significant difference between the preoperative and postoperative BCVA (*P* < 0.001) [22]. Strict control of the preoperative inflammation was shown to significantly improve the visual outcomes of cataract surgerywith IOL implantation [23]. In this study, patients with trematode anterior uveitis showed better visual results than other patients with uveitis because strict preoperative control of inflammation is possible with less liability to recurrences after control of the disease compared to other uveitic entities especially autoimmune uveitis, less severe pre-existing vision-limiting pathology, and less postoperative inflammation.

Patients with complicated cataracts are expected to experience a higher incidence of postoperative complications than patients with age-related cataracts.Moreover, reactivation of uveitis after surgery leads to a higher risk of development of complications and may contribute to unfavourable outcomes. [24]. In our study, the most common postoperative complications were PCO (two patients developed PCO) and reactivation of uveitis; however, there was no elevation of intraocular pressure (IOP) or CME. A previous study reviewed 17 patients who underwent coaxial micro-incision cataract surgery and reported that the occurrence of postoperative complications was rare; IOP elevation and uveitis exacerbation were observed in one eye [25].

In this current study, no statistically significant difference was observed between the preoperative and postoperative endothelial cell count. Our results suggest that perioperative systemic therapies and recurrent uveitis are important risk factors for postoperative complications such as PCO, CME, and elevated IOP. In contrast, previous studies have indicated that the administration of prophylactic corticosteroids is associated with decreased incidences of Nd: YAG capsulotomy and CME [26].

There are some limitations to our study, namely the small sample size, retrospective design and the short follow-up period.

In conclusion, this study showed that most patients with trematode-induced anterior uveitis experienced significant improvement in visual acuity after cataract surgery compared to patients with other uveitis entities. Patients with relapse inflammation were at an increased risk of developing postoperative complications, including PCO and CME. Future extensive randomized, double-blind prospective studies are warranted to demonstrate the factors predisposing to devvelopment of surgery complications.

## Data Availability

All data generated or analyzed during this study are included in this article. Further inquiries can be directed to the corresponding author.

## Abbreviations

BCVA: Best-corrected visual acuity
AC: Anterior chamber
CCC: Continuous curvilinear capsulorhexis
OVD: Ophthalmic viscoelastic device
IOL: Intra ocular lens
HMGP: Hand motion good projection
OCT: Optical coherence tomography
CMT: Central macular thickness
CME: Cystoid macular edema
PCO: Posterior capsular opacification
ECC: Endothelial cell count
